# On the Use of Anæsthetics in Midwifery

**Published:** 1849-05

**Authors:** J. Y. Simpson

**Affiliations:** Edinburgh


### MEDICAL EXAMINER,

AND

### RECORD OF MEDICAL SCIENCE.

NEW SERIES.—NO. LIII.—MAY, 1849.

---

### ORIGINAL COMMUNICATIONS.

---

### *On the use of Anæsthetics in Midwifery.* By J. Y. SIMPSON, M. D., of Edinburgh.

[Concluded from page 218.]

4. *You object to anæsthesia in labour, because the mother in escaping by it from the "pangs and agonies of labour" may in a few rare cases be made to encounter danger to her own life.*

"Should I (you observe) exhibit the remedy for pain to a thousand patients in labour, merely to prevent the physiological pain, and for no other motive, and if I should, in consequence, destroy only one of them, I should be disposed to clothe me in sackcloth and cast ashes on my head for the remainder of my days. What sufficient motive have I to risk the life or the death of one in a thousand in a questionable attempt to abrogate one of the general conditions of man?" Let me add, that I have seen this argument of yours already repeated from your letter, and strongly insisted upon by the opponents of anæsthesia in this country.

And, indeed, in a new practice, such as that of anæsthesia, and with which the mind is not yet at all familiarized, the above forms one of that kind of apparently strong statements which it is impossible to answer directly, or indeed by any other way than by taking, as I have already said, a corresponding illustration and simile from some other matter with which the mind is already familiarized. Let us for a moment longer, then, adhere to the familiar comparison which I have already taken up, under the last head, between the physiological function of human parturition and the physiological function of human progression. Suppose, then, that you and I were standing at the Philadelphia station on the first day of opening of the railway to Baltimore or New York. I wish the passengers to Baltimore or New York, or the shorter or intermediate stations, to proceed thither by railway; but you argue with them, like President Jefferson, that "progression is the culminating point of the human somatic forces," and that "walking is a desirable, salutary, and conservative manifestation of life force," and that progression being a "physiological function," and fatigue a physiological pain, they ought to proceed on foot. I say "No." Place yourself in a railway carriage, and thus eschew and obviate all the great fatigue and useless over exertion of foot travelling. Then comes that answer and argument of yours which I have quoted, and which runs as follows; "But should I exhibit, sir, the remedy for fatigue (*a railway carriage*) to a thousand travellers, merely to prevent the physiological exertion and fatigue of walking, and for no other motive, and if I should in consequenee destroy only one of them, I should feel disposed to clothe me in sackcloth and cast ashes on my head for the remainder of my days. What sufficient motive have I had to risk the death of one in a thousand in a questionable attempt to abrogate one of the general conditions of man, viz., his power of walking."

I shall not stop to enquire whether among our supposed lady passengers or patients (unused as most of them are either to long pain or long walking) more than one in a thousand would not be worn out and destroyed by taking the journey on foot. A less proportion would, I believe, be found to be ultimately destroyed by the perils and dangers of the journey by railway, than by the exertion and fatigue of the journey on foot, and the walk would shake and damage, both temporarily and permanently, many more constitutions than the railway carriage. I have a firm conviction, that on the great scale, there would be found a more absolute saving of human life and human health by adopting the means invented by art than the means provided by nature. And I most firmly believe that yet a similar difference will be found to hold good between the two corresponding practices of allowing women to pass through labour afflicted with all their usual physiological pangs and agonies, and carrying them through that process without being subjected to the endurance of these pangs and agonies.

But I proceed to remark, that if your supposed theory with regard to the function of parturition were carried out in regard to the other functions of the human body, it would produce a vast and mighty revolution in the practices of civilized life. Follow it out, for instance, with regard to any one of them, as for example with regard to the one we have already spoken of, viz. progression, and see what would be the result. Ever and anon, our newspapers contain paragraphs telling us of one or more human lives being lost by collisions on railways, explosions on steamboats, upsettings of stage coaches, &c. Consequently, according to your doctrine, the featherless biped, pedestrian man, should no longer, when travelling, fly in railway cars, ply in steamboats, ride in coaches, &c., for these are evidently all so many questionable attempts to abrogate, what you call "one of the general conditions of man, viz., his original pedestrianism."

In the great government and police of nature, disease and death are among the most certain and "general conditions of man." If your theory were true, the practice of medicine itself should, I fear, be at once and summarily abandoned, for perhaps, in your own language, it is at least "a qustionable attempt to abrogate one of the general conditions of man," and I am sure you will agree with me that in this questionable attempt "human lives are often lost from the mistakes, or the passiveness, or the want of knowledge and skill on the part of the physician. In England and Wales in 1840, there were, according to the Returns of the Registrar General, above 100 persons publicly and officially reported as having died from the effects of one drug alone—opium. But would this be any reason or any ground of reason for abandoning in medicine the use of opium, perhaps in itself the most valuable of all the remedies in our Pharmacopœia. Would this be any adequate argument for refusing to relieve by a dose of opium the next appropriate case of pain that you are called to? Or because chloroform or ether in a very rare case now and again produces deleterious or more fatal consequences, should we refuse in a thousand other persons to mitigate and annul their agonies by its use?

In your esteemed letter to me you quote some remarks from that celebrated old work—Raynolde's Birthe of Mankinde—the first book on midwifery printed in English. Look at the Prologue to the work. It is in reference to the very subject we are discussing; whether the rare accidents, from abuse or otherwise, to which any good gift may occasionally subject those who use it, should be a reason for repudiating the general use of that gift. "There is not anything," says Raynolde, "so absolute and perfect but by the occasion of the abuse thereof at one time or other, may, and doth ensue, great damage and danger to mankinde." He instances fire and water, "two right necessary elements to the use of man, without the which he could not live;" yet sometimes "by fire hath bin consumed and devoured whole cities and countries; by water swallowed and drowned infinite men, shippes, yea and whole regions. Againe, (he continues,) meate and drinke to the moderate users thereof, doth minister and maintain life; and contrary to the unmeasurable and unsatiate gourmands and gluttons it hath full many times brought surfeit and sickness, and at the last death. But (he argues) should men for the avoyding of the aforesaid inconveniences and for the reasons above said, condemne and banish fire and water or forsake their meate and drinke? No. *It were but madnesse once to thinke it.*"

Before passing from these your supposed dangers of anæsthetics, let me add two remarks. 1st. I do believe that if improperly and incautiously given, and in some rare idiosyncrasies, ether and chloroform may prove injurious or even fatal, just as opium, calomel, antimony, and every other strong remedy and powerful drug will occasionally do. Drinking cold water itself will sometimes produce death. "It is well known that there are *many* cases," says Dr. Taylor in his excellent work on Medical Jurisprudence, "on record, in which cold water swallowed in large quantity and in an excited state of the system, has led to the destruction of life." (p. 8.) Should we therefore never allay our thirst with cold water? What would the disciples of Father Mathew say to this? But 2dly. You and others have very unnecessary and aggravated fears about the dangers of ether and chloroform, and in the course of experience you will find these fears to be perfectly ideal and imaginary. But the same fears have in the first instance been conjured up against almost all other innovations in medicine, and in the common luxuries of life. Revert again to our old simile regarding travelling. Cavendish, the Secretary to Cardinal Wolsey, tells us in his life of that prelate, that when the Cardinal was banished from London to York by his master, that regal Robespierre, Henry VIII., many of the Cardinal's servants refused to go such an enormous journey, for they were (says Cavendish) "loath to abandon their native country, their parents wives and children." The journey which can now be accomplished in six hours, was considered then a perfect banishment. We travel now between London and Edinburgh (some 400 miles) in twelve or fourteen hours. A century ago, the stage coach took twelve or fourteen days. And in his life of Lord Loughborough, Lord John Campbell tells us, "that when he (the biographer) first travelled from Edinburgh to London in the mail coach, the time was reduced to three nights and two days; but he adds, this new and swift travelling, from the Scotch to the English capitol, was wonderful, and I was gravely advised (adds Lord John) to stop a day at York, as several passengers who had gone through without stopping had died of apoplexy from the rapidity of the motion." (*Lives of the Lord Chancellors*, vol. vi. p. 50.)

Be assured that many of the cases of apoplexy, &c., &c., alleged to arise from ether and chloroform, have as veritable an etiology as this apoplexy from rapid locomotion; and that a few years hence they will stand in the same light in which we now look back upon the apoplexy from travelling at the rate of ten miles an hour. And as to the supposed great moral and physical evils and injuries arising from ether and chloroform, they will bye and bye sound, I believe, much in the same way as the supposed great moral and physical evils and injuries arising from using hackney coaches, as all were seriously described by Taylor, the water-poet two or three centuries ago, when these coaches were first introduced. In his diatribe against hackney coaches, Taylor warned his fellow creatures to avoid them, otherwise, to quote his own words, "they would find their bodies tossed, tumbled, rumbled, and jumbled without mercy." "The coach (says he) is a close hypocrite, for it hath cover for knavery; they (the passengers) are carried back to back in it like people surprised by pirates, and, moreover, it maketh men imitate sea-crabs in being drawn sideways," and altogether "it is a dangerous carriage for the commonwealth." Then he proceeds to call them "hell carts," &c., and vents upon them a great deal of other abuse very much of the same kind and character as that lavished against anæsthetics in our own day.

In the course of your remarks you imply, I think, though you nowhere explicitly state, another objection to anæsthetics in midwifery, viz :

5. *You object to anæsthesia in labour because you do not consider that the mother encounters danger to her health or life from the endurance of the pains.*

"I have been accustomed (you observe) to look upon the sensation of pain in labour as a physiological relative of the power or force, and, notwithstanding I have seen so many women in the throes of labour, I have always regarded a labour-pain as a most desirable, salutary conservative manifestation of life-force."

If you hold, as your language appears to me to imply, that the sensation of pain, even when, as in labour, the degree of the pain is "absolutely indescribable," has no morbid or deleterious influence upon those who endure it, then I most decidedly disagree with you. On the contrary, I sincerely believe that the human constitution is so constituted that it cannot endure pain, particularly when that pain is long in duration or severe in degree, without being more or less affected and injured by it. I know of many medical and obstetric authors from the time of Ambrose Paré, down to the time of Travers, Gooch, Alison, Burns, &c., who have stated and explained the common and hitherto unchallenged opinion of our profession in all ages, that pain was, in itself, deleterious and destructive, causing depression of the heart, syncope, and even when in excess producing speedy and sudden death. But till the late discovery in your own country, of the possibility of annulling the pains of surgical operations by the inhalation of ether, I know of no writer in medicine or surgery or in midwifery, who held that pain when "absolutely indescribable" in degree, was a matter of no importance in regard to the life or health of the sufferer, and should not be relieved even when we had the complete power of relieving it.

If the mere pain of the labour were, as you state, a "desirable, salutary, and conservative manifestation of life-force," its long continuance, the very length of it, would ensure more certainly the life and health of the patient than its shortness. Anything "salutary and conservative" to the constitution, should manifestly be safe in proportion to the length and dangerous in proportion to the shortness of its duration. But as far as regards the life and health of the mother, the pain of labour is perfectly the reverse of all this. It is safe in proportion to its shortness, and dangerous in proportion to its length. In the Dublin Hospital, the tables of which afford the only data on this point that I know to refer to, when the women were four hours in labour, more subsequently died than when their pain did not exceed two hours; of those that are eight hours in labour, more subsequently died than of those that were four hours ill; of those that were twelve hours in suffering, more subsequently died than of those that were eight, and so on in a regular progression. The longer this supposed "salutary and conservative manifestation of life-force," as you term it, the greater became the mortality, so that in the long run the maternal mortality was fifty times greater among the women that were above thirty-six hours ill, than among those who were only two hours in labour; one in every six of the former dying in child-bed; and one only out of every three hundred and twenty of the latter.

Some time ago I published a long series of statistics tending to show, that out of a large collection of cases of the same operation performed with and without anæsthesia, those who were operated on under anæsthesia, and consequently without the usual sufferings, recovered in a much larger proportion than those who had the same operation performed without anæsthesia, and whose constitutions were subjected to the endurance of the usual pains and agonies of the surgeon's knife.

The same result holds good, I believe, in midwifery as in surgery. Save the maternal constitution, either by natural or artificial anæsthesia, from the endurance of the pains connected with parturition, and you will enhance both the chances of her recovery and the facility of it. Among your red Indian and other uncivilized tribes, the parturient female does not suffer the same amount of pain during labour as the female of the white race; and in consequence of this escape, they recover far more rapidly from the effects of parturition, nor are fatalities at all common among them. So easy is the convalescence among uncivilized tribes, that Strabo, Marco Polo, and other historians and travellers, tell us of whole communities in which the husband immediately went to bed upon the birth of a child, and the wife watched and nursed him. "They that wrote the history of America (says Guillemeau) tell of the women in that country, that as soon as they be delivered, they presently rise up and lay their husbands in their roome, who are used and attended like women in child-bed."

Among the patients who have been delivered in Scotland under anæsthesia, the rapidity of the stage of convalesence has, as a general rule, been diminished in a degree that seems often to surprise the patient herself, as much as her escape from the labour pains themselves. Many of my obstetric brethren have remarked this circumstance to me. In fact, on awaking after delivery, the patient does not encounter and endure the usual feelings of exhaustion and fatigue. Some have declared to me that they have felt as if they had awoke from a refreshing sleep. And when we consider the capabilities for the enduring of suffering and exertion among the class of persons in civilized life upon whom you and I attend, perhaps the propriety of employing anæsthesia during labour may appear more evident. Unaccustomed by their mode of life to much pain and fatigue, patients in the higher ranks of life are not fitted to endure either of them with the same power or with the same impunity as the uncivilized mother, or even as females in the lower and hardier grades of civilized society, and hence there is the greater propriety and necessity in the physician employing all the means of his art so as to save them, as far as possible, from their sufferings. To illustrate the point, let us revert again to our old comparison between the physiological functions of progression and parturition. Let us compare, for a moment, our ideas of the effects of fatigue from walking, and of pain from parturition, upon the female constitution; and surely the comparison is not an unfair one for your views, as far as the severity of the effects of the two influences, physical fatigue and physical pain, are concerned, for surely the effects of pain, of absolutely indescribable pain, should be greater upon the constitution than mere muscular fatigue. Suppose, then, that our patients at the end of the ninth month of pregnancy, had to walk on foot a continuous journey of one, two, three, six or a dozen or more hours' duration, that is of five, ten, twenty, thirty miles or upwards, instead of passing through a continuous journey of recurring labour pains of the same duration, the pains gradually becoming stronger, and latterly becoming "absolutely indescribable and comparable to no other pains;" what would be the result with say one hundred ladies of the upper classes of society? Some of them might be little or not at all affected by the journey, others, weak perhaps when they began, would suffer more or less severely from it. Not a few would be inclined sooner or later to stop and beseech you, if you were the medical attendant upon them, to save them from further exertion and fatigue, by allowing them to be carried or coached the required distance. In answer to their solicitations would you console them by telling them that, after all, progression was a "conservative manifestation of life-force," and free from danger, or would you take the other view and give them means of travelling the required distance by carriage or rail?

I am sure you would have recourse not to the former but to the latter, for you would fear and dread the effects of fatigue upon the fragile constitutions of your lady patients. And I repeat, that certainly the effects of the endurance of pain are as great, if not greater upon the constitution than the effects of the endurance of fatigue. But if you would allow your patients to ride the supposed journey, instead of unnecessarily forcing and compelling them to walk it on foot; equally I think should you allow them to escape what you term the "pangs and agonies" of travail, by saving them by chloroform or other anæsthetic agents during their travail, from all the unnecessary endurance of these pangs and agonies.

You state, "I have not yielded to several solicitations as to the exhibition [of chloroform] addressed to me by my patients in labour." If, when driving out into the country, you perchance met one of your fair patients a few miles from Philadelphia, walking homeward, but so tired and way-worn, that every five or ten minutes she stopped and groaned for fatigue "absolutely indescribable and comparable to no other fatigue," I am sure you would consider yourself bound on the principles of common humanity not to withstand her "solicitations" to be driven home in your carriage, and thus relieved of her present anxiety and suffering. And I cannot see why, if you do this, (and who would not do it?) to relieve a patient from the mere effects of fatigue, you could refuse to relieve the same lady when in the "pangs and agonies of travail," from the endurance of pains which, in your own words, are "absolutely indescribable and comparable to no other pains."

"Perhaps (you observe) I am cruel in taking so dispassionate a view of the subject." Of course it would ill become me to pass any such judgment upon you. But I feel this, that you and I and other teachers of midwifery are placed, in reference to this question, in a position far more fearfully responsible than ordinary medical practitioners. The ordinary obstetric practitioner has little or no power, except over the relief or the perpetuation (according as he may choose it) of the sufferings of his own immediate patients. But you and I, as obstetrical teachers, may, through our pupils, have the power of relieving or of continuing the sufferings of whole communities. If, perchance, you persist for some years longer in your present opinion, it will have the effect of inflicting a large amount of what I conscientiously believe and know to be altogether unnecessary agony and suffering, upon thousands of our fellow beings. If you review and alter your opinions, (which I earnestly hope you will do,) and make yourself sufficiently acquainted with the peculiarities in the mode of action, in the mode of exhibition of chloroform during labour, a vast proportion of human suffering may, even within the next few years, be saved by your happy instrumentality and influence.

Feeling, as I do, deeply, the great responsibility in this respect of your situation and of mine, I trust you will kindly pardon and excuse me, if, anywhere in the preceding pages, I may have appeared to defend my views with too much earnestness. If I had to rewrite or revise the observations, I would perhaps have stated them more accurately, but I must send them as they are. And along with them I beg to send also the most sincere esteem and reiterated respects of

My dear sir,

Yours very faithfully,

J. Y. Simpson.

To Dr. Meigs, Professor of Midwifery, Philadelphia.

